# Perceptions and Practice of Urologists in Saudi Arabia Regarding Sexual Complications Related to LUTS/BPH Management

**DOI:** 10.3390/jcm14124367

**Published:** 2025-06-19

**Authors:** Saad Alshahrani, Abdulrahman Binsaleh, Ahmed Othman Alghamdi, Saad Alqasem, Ali Al-Gonaim, Ashraf El-Metwally

**Affiliations:** 1 Department of Surgery, Division of Urology, College of Medicine, Prince Sattam bin Abdulaziz University, Al-Kharj 11942, Saudi Arabia; abd.binsaleh8@gmail.com (A.B.); ahmad.oth.g@gmail.com (A.O.A.); s.alqasem@psau.edu.sa (S.A.); a.algonaim@psau.edu.sa (A.A.-G.); 2 College of Public Health and Health Informatics, King Saud bin Abdulaziz University for Health Sciences, Riyadh 11426, Saudi Arabia; elmetwally.ashraf@outlook.com; 3 King Abdullah International Medical Research Center, Riyadh 11481, Saudi Arabia

**Keywords:** benign prostatic hyperplasia, sexual dysfunction, urologists, perceptions, Saudi Arabia

## Abstract

**Objectives**: This study aimed to evaluate perceptions and practices of urologists in Saudi Arabia regarding discussions of erectile dysfunction (ED) and ejaculatory dysfunction (EjD) with patients before initiating BPH treatments. **Methods**: A cross-sectional survey was conducted using a structured questionnaire distributed during the 36th Saudi Urological Annual Conference held in Riyadh in February 2025 among urologists in Saudi Arabia. A binary outcome variable, “frequent and open discussion,” was created based on a scoring system using the median score of these responses. Data analysis included descriptive statistics and univariate (*p* < 0.25) and multivariate (*p* < 0.05) logistic regression using SPSS version 27. **Results**: Discussions about ED risks were most frequent before prescribing 5-alpha reductase inhibitors (5-ARIs) (51.3%) and combined alpha-blockers and 5-ARIs therapy (50.0%), whereas EjD risks were more frequently addressed before alpha-blocker monotherapy (59.2%) and transurethral resection of the prostate (TURP) (56.6%). A substantial proportion of urologists discussed alternative treatments based on sexual dysfunction risks, particularly before TURP (53.9%), alpha-blockers (47.4%), and 5-ARIs (43.4%). Univariate analysis revealed a trend towards more open discussions among non-Saudi urologists (OR 4.58, 95% CI 0.88–23.74, *p* = 0.06) and a significant association with working in private hospitals (OR 3.68, 95% CI 0.39–35.14, *p* = 0.03). However, these associations did not hold in multivariate analysis. **Conclusions**: Urologists in Saudi Arabia demonstrate variability in discussing sexual complications with patients before BPH treatments. Consistent and comprehensive discussions about ED and EjD risks are crucial for informed patient decision-making. Standardized guidelines and educational programs are needed to enhance urologists’ communication skills and ensure consistent patient counseling.

## 1. Introduction

Benign prostatic hyperplasia (BPH) is a common age-related condition that significantly impacts men’s quality of life due to lower urinary tract symptoms (LUTSs) [1,2]. Management of LUTS/BPH includes a range of options, from medical therapies like alpha-blockers and 5-alpha reductase inhibitors (5-ARIs) to surgical interventions such as transurethral resection of the prostate (TURP) and holmium laser enucleation of the prostate (HoLEP) [3,4,5]. Despite their efficacy in alleviating urinary symptoms, these treatments often lead to sexual complications, including erectile dysfunction (ED), ejaculatory dysfunction, and decreased libido, which can profoundly affect patients’ well-being [6,7,8]. Sexual dysfunction is a well-documented side effect of BPH treatment, with each modality carrying distinct risks [6,7,8]. Alpha-blockers may cause ejaculatory dysfunction (EjD), while 5-ARIs can lead to decreased libido and ED [9]. Surgical procedures like TURP and HoLEP can result in retrograde ejaculation and, in some cases, ED [10].

The impact of BPH treatment on sexual function is a critical concern for patients, influencing their treatment choices [11,12]. Cultural attitudes and patient expectations further complicate the management of sexual health in medical consultations [13]. In conservative societies like Saudi Arabia, discussions about sexual function may be particularly challenging due to social taboos and cultural sensitivities [14]. Urologists play a vital role in counseling patients about these risks, but their perceptions and practices in discussing and managing sexual complications are not uniform and are not explored widely in the literature. More precisely, there is a lack of studies, especially in countries such as Saudi Arabia, exploring the perceptions and practices regarding sexual dysfunction discussions in BPH management. Therefore, this study aims to evaluate the frequency and method followed by urologists in Saudi Arabia in discussing the possibility of ED and EjD with patients before initiating various treatments for BPH. By exploring these aspects, this research seeks to provide valuable insights into how sexual health is addressed in the context of BPH management in Saudi Arabia and to highlight areas for improvement in clinical practice.

## 2. Materials and Methods

### 2.1. Study Design

This study employed a cross-sectional, survey-based design to investigate the perceptions and practices of urologists in Saudi Arabia regarding the discussion of sexual complications related to BPH management. The cross-sectional approach was chosen to provide a snapshot of current practices and attitudes at a specific point in time. The survey was planned for distribution during the 36th Saudi Urological Annual Conference in Riyadh (2025), a key event that attracts a significant number of practicing urologists from across the country, ensuring a broad representation of the target population.

### 2.2. Study Population and Eligibility Criteria

The target population for this study consisted of urologists working in both public and private hospitals and clinics throughout Saudi Arabia. This diverse setting allowed for the inclusion of a wide range of perspectives and experiences. Practicing urologists at various career levels, including residents, fellows, specialists, and consultants, who are involved in the management of BPH patients were included in the study. Urologists who do not manage BPH patients were excluded to ensure that the survey responses were relevant to the study’s focus.

### 2.3. Study Questionnaire and Data Collection

Data collection was conducted using a structured, self-administered questionnaire designed to gather comprehensive information regarding urologists’ perceptions and practices related to discussing sexual complications with patients undergoing BPH treatment. Data collection was conducted using a structured questionnaire, which was distributed during the 36th Saudi Urological Annual Conference held in Riyadh in February 2025. The questionnaire consisted of two main sections. The first section focused on gathering demographic and professional information, including nationality, working institution such as public hospital, private clinic, or education–research hospital, professional status such as resident, fellow, specialist, or consultant, and workload, assessed by the number of patients seen per clinic session and the number of LUTS/BPH cases managed per month. The second section explored urologists’ approaches to discussing sexual complications with patients before initiating BPH treatment, including the frequency with which urologists discuss specific sexual dysfunctions, namely ED and EjD. These discussions were assessed in the context of various medical and surgical treatments for BPH, including alpha-blockers, 5-ARIs, transurethral resection of the prostate, photoselective vaporization of the prostate, Rezum water vapor therapy, HoLEP, and combined therapy of alpha-blockers and 5-ARIs. The questionnaire utilized a combination of question formats, including multiple-choice questions, Likert scales, and open-ended questions, to capture both quantitative and qualitative data, allowing for a comprehensive assessment of urologists’ practices and perceptions, and providing a detailed understanding of the current state of sexual health discussions in BPH management within Saudi Arabia.

### 2.4. Data Analysis

Descriptive statistics were utilized to summarize the perceptions and practices of urologists regarding the discussion of sexual complications in BPH management. For questions regarding the frequency of discussions about sexual dysfunction, responses were categorized as “Always,” “Often,” “Sometimes,” “Rarely,” “Never,” and “Not Applicable” (N/A). To facilitate regression analysis, a scoring system was developed, assigning higher scores to responses indicating more frequent discussions (e.g., “Always”) and lower scores to responses indicating less frequent discussions (e.g., “Never”). Using the median score, a binary outcome variable was created, categorizing responses into “frequent and open discussion” versus “less frequent discussion.”

Univariate logistic regression analysis (using a *p*-value cutoff of <0.25) was performed to explore potential predictors of frequent and open discussions about sexual dysfunction. Subsequently, multivariate logistic regression analysis (using a *p*-value cutoff of <0.05) was conducted to identify independent predictors, adjusting for potential confounders. Odds ratios (ORs) and 95% confidence intervals (CIs) were calculated to assess the strength of associations. All statistical analyses were performed using SPSS version 27. A *p*-value of <0.05 was considered statistically significant, unless otherwise specified for the univariate analysis.

## 3. Results

### 3.1. Demographic and Practice Characteristics of Urologists

Table 1 presents the demographic and practice characteristics of urologists working across Saudi Arabia. The professional status of the respondents was varied, with residents comprising the largest group (51.3%), followed by consultants (18.4%) and specialists (22.4%). Fellows made up the smallest proportion (7.9%). Most of the urologists surveyed were Saudi nationals (88.2%), with the remaining 11.8% being non-Saudi. Geographically, most of the participants worked in the Central Region (56.6%), while other regions were represented to a lesser extent, including the Eastern Region (14.5%), South Region (11.8%), Western Region (10.5%), and North Region (6.6%). In terms of practice volume, a significant proportion of urologists (56.6%) reported seeing more than 15 patients per clinic, while 26.3% saw 10–15 patients, and 17.1% saw fewer than 10. The institutions where these urologists practiced varied, with the highest proportion working in public hospitals (47.4%), followed by education–research hospitals (38.2%) and private hospitals (14.4%). Finally, regarding their caseload of LUTS and BPH, 39.5% of urologists reported seeing over 30 cases per month, while 25% saw 6–15 cases, 21.1% saw 16–30 cases, and 14.5% saw 0–5 cases, as shown in Table 1. Regarding treatment options offered by urologists for LUTS and BPH, the most common approach was a combination of alpha-blockers, 5-ARIs, and TURP, accounting for 19.7% of responses. Additionally, a significant proportion (17.1%) of urologists utilized alpha-blockers, 5-ARIs, TURP, and Rezum as a treatment strategy.

### 3.2. Discussion of Sexual Dysfunction Risks Before Treatment

Table 2 presents the frequency and percentage of how often urologists discuss ED and EjD risks with their patients before prescribing various treatments for LUTS/BPH.

#### 3.2.1. Discussion of ED with Patients Before Prescribing Treatment

Urologists most consistently discuss the risk of ED with patients before prescribing alpha-blockers and 5-ARIs, with over 40% reporting they “always” have this discussion, as shown in Table 2. For TURP, approximately one-third of urologists “always” discuss ED risks. In contrast, discussions about ED risks are less frequent for PVP, HoLEP, and Rezum treatments. However, it is worth noting that a considerable proportion of respondents discuss ED risks “sometimes”: percentages of 15.8% for PVP, 14.5% for HoLEP, and 28.9% for Rezum. Additionally, some urologists indicated that they discuss ED risks “rarely”: percentages of 14.5% for PVP, 15.8% for HoLEP, and 13.2% for Rezum (Table 2). This suggests that while routine “always” discussions are less common for these treatments, the potential for ED is still addressed with patients, albeit on a less consistent basis.

#### 3.2.2. Discussion of EjD with Patients Before Prescribing Treatment

The risk of EjD is most frequently discussed before prescribing alpha-blockers and TURP, with over 65% of urologists reporting they “always” have this conversation, as illustrated in Table 2. Discussions about EjD risks are also common before prescribing 5-ARIs, with 35.5% reporting “always” discussions. For PVP, HoLEP, and Rezum, discussions about EjD are less consistently reported as “always,” with a notable proportion of respondents reporting discussions “sometimes” (19.7%, 15.8%, and 23.7%, respectively). Additionally, a significant proportion report discussing EjD risks “rarely” for HoLEP (10.5%) and Rezum (9.2%). This suggests that while these treatments are not discussed always, EjD risks are still addressed in a significant number of cases, albeit less consistently, through “sometimes” or “rarely” discussions, likely based on patient-specific factors.

### 3.3. Discussion or Counseling of Alternative Treatments

#### 3.3.1. Discussion of Alternative Treatments Based on Sexual Dysfunction Risks

Table 3 illustrates the frequency with which urologists discuss alternative treatments or counsel patients regarding them, based on the risk of sexual dysfunction associated with various therapies for LUTS/BPH. Before prescribing TURP, 5-ARIs, and alpha-blockers, a significant proportion of urologists “always” discuss alternative treatment options, with approximately 47.4%, 47.4%, and 40.8%, respectively, consistently engaging in this discussion. Similarly, discussions of alternatives “always” occur in 28.9% of Rezum cases, 23.7% of PVP cases, and 19.7% of HoLEP cases. Discussions about alternative treatments happen “sometimes” across all treatments, with a similar proportion of Rezum, alpha-blockers, and TURP cases reporting “sometimes” discussions (23.7%, 23.7%, and 25%, respectively). The N/A response is highest for HoLEP and PVP, suggesting that some urologists may not consider discussing alternative treatments relevant in these cases or they do not perform those procedures in their institutions.

#### 3.3.2. Alternative Treatment Counseling Based on Sexual Dysfunction Risks

This table presents the frequency with which urologists discuss alternative treatments with their patients based on the risks of sexual dysfunction associated with various therapies for LUTS/BPH. A substantial proportion of urologists report “always” discussing alternative treatments before prescribing TURP (53.9%), alpha-blockers (47.4%), and 5-ARIs (43.4%), indicating a consistent practice of addressing sexual dysfunction risks. For Rezum, 35.5% of urologists “always” discuss alternatives, while 22.4% do so for HoLEP and 21.1% for PVP. Discussions about alternative treatments happen “sometimes” across all treatments, with a similar proportion of Rezum, alpha-blockers, and TURP cases reporting “sometimes” discussions (27.6%, 21.1%, and 21.1%, respectively). The N/A response is highest for HoLEP (34.2%) and PVP (38.2%), suggesting that some urologists may not consider discussing alternative treatments relevant in these cases, possibly due to perceived lower risks of sexual dysfunction or patient-specific considerations or because they do not perform those procedures in their institutions.

### 3.4. Urologists’ Discussion of ED and EjD Before Starting Treatment

Table 4 presents the frequency with which urologists discuss the possibility of ED with patients before starting various treatments for LUTS/BPH:

#### 3.4.1. Discussion of ED Before Starting Treatment

As shown in Table 4, discussions about ED risks “always” occur most frequently before starting 5-ARIs (51.3%) and the combined alpha-blocker and 5-ARI therapy (50.0%). Before TURP, 38.2% of urologists report “always” discussing ED risks. For Rezum and alpha-blocker monotherapy, 30.3% of urologists report “always” discussing ED. Discussions about ED risks are least frequent before PVP, with only 19.7% of urologists reporting “always” discussions. A significant proportion of urologists’ report discussing ED risks “sometimes” or “often” across all treatments. Notably, the “N/A” response is highest for PVP (36.8%), suggesting that some urologists may not consider discussing ED risks relevant in these cases, possibly due to perceived lower risks of ED or patient-specific considerations or because they do not perform the procedure in their hospitals.

#### 3.4.2. Discussion of EjD Before Starting Treatment

Table 4 illustrates the frequency with which urologists discuss the possibility of EjD with patients before initiating various LUTS/BPH treatments. A significant proportion of urologists consistently “always” discuss EjD risks before prescribing alpha-blocker monotherapy (59.2%), TURP (56.6%), and the combined alpha-blocker and 5-ARI therapy (47.4%). Discussions about EjD risks “always” occur in 39.5% of 5-ARIs cases and 31.6% of Rezum cases. The lowest frequency of “always” discussions is observed for HoLEP (22.4%) and PVP (14.5%). Notably, the “N/A” response is highest for HoLEP (34.2%) and PVP (39.5%), suggesting that some urologists do not perform those procedures or due to perceived lower risks or patient-specific factors. Across all treatments, a considerable number of urologists report discussing EjD risks “often” or “sometimes,” indicating a general awareness and consideration of this potential side effect.

### 3.5. Predictors of Frequent and Open Discussion About Sexual Dysfunction

Table 5 illustrates the findings on the predictors of frequent and open discussion about sexual dysfunction. More precisely, univariate analysis, using a *p*-value cutoff of 0.25, revealed several factors with potential associations with the frequency and openness of discussing sexual dysfunction. Non-Saudi nationality showed a trend towards more frequent and open discussions compared to Saudi nationals (OR 4.58, 95% CI 0.88–23.74, *p* = 0.06). The type of working institution demonstrated a significant association (*p* = 0.03). Specifically, urologists working in private hospitals (OR 3.68, 95% CI 0.39–35.14) were more likely to have frequent and open discussions compared to those in education–research hospitals, while those in public hospitals (OR 0.35, 95% CI 0.13–0.94) were less likely. The number of LUTS/BPH cases seen per month also showed a trend towards association, with higher odds of frequent discussion observed for those seeing 16–30 cases (OR 1.43, 95% CI 0.32–6.46; *p* = 0.22).

In the multivariate analysis, using a *p*-value cutoff of 0.05, none of the factors achieved statistical significance. While the trend for non-Saudi nationality persisted with a notably higher odds ratio (AOR 7.28, 95% CI 0.69–76.88, *p* = 0.09), it did not meet the significance threshold. Multivariate analysis could not be performed for profession and region. The working institution, which was significant in the univariate analysis, did not remain significant in the multivariate model (*p* = 0.25), although the point estimates continued to suggest a higher likelihood of frequent discussion in private hospitals (AOR 3.14, 95% CI 0.20–50.04) and a lower likelihood in public hospitals (AOR 0.43, 95% CI 0.12–1.61) compared to education–research hospitals. Similarly, the trend observed for the number of LUTS/BPH cases per month in the univariate analysis did not reach statistical significance in the multivariate model (*p* = 0.38), with the highest odds ratio observed for those seeing 16–30 cases (AOR 2.79, 95% CI 0.26–29.68).

## 4. Discussion

This study aimed to evaluate the perceptions and practices of urologists in Saudi Arabia regarding the discussion of sexual complications with patients before initiating various treatments for LUTS/BPH. Our findings revealed variations in the frequency and manner in which urologists discuss ED and EjD risks with patients across different BPH treatment modalities. Notably, discussions about ED risks were most consistent before prescribing 5-ARIs and combined alpha-blockers and 5-ARIs therapy, while EjD risks were more frequently addressed before alpha-blockers monotherapy and TURP. We also observed that a substantial proportion of urologists consistently discuss alternative treatments based on the risk of sexual dysfunction, particularly before prescribing TURP, alpha-blockers, and 5-ARIs. Predictors of frequent and open discussion about sexual dysfunction were explored, revealing a trend towards more open discussions among non-Saudi urologists and a significant association with working in private hospitals in the univariate analysis, though these associations did not hold in the multivariate model.

Comparing our findings with those reported by Giona et al. (2018), we observed both similarities and differences [15]. Like their study, we found variations in the frequency with which urologists discuss ED and EjD risks. However, while their study reported that approximately 70% of specialists discuss ED before prescribing alpha-blockers (which are not typically known to cause ED), our study found that ED discussions were more prevalent before 5-ARIs and combined therapy [15]. Regarding EjD, both studies found that discussions were common before alpha-blockers and TURP [15]. Our study further specified that discussions were also frequent before 5-ARIs. Both studies highlight that a significant minority of urologists do not consistently discuss these complications.

Furthermore, both studies observed that many respondents do not routinely discuss alternative therapies based on the risk of sexual dysfunction. Notably, Giona et al. (2018) found that urologists with higher caseloads were less likely to offer alternative therapies, a trend that, although not specifically assessed in our study, underscores the potential impact of workload on counseling practices [15]. It is important to acknowledge that direct comparisons are challenging due to the limited number of studies specifically examining urologists’ counseling practices regarding sexual dysfunction in BPH management. The variability in study designs, participant demographics, and cultural contexts further complicates the comparison. Nonetheless, both studies underscore the need for improved counseling practices and highlight the discrepancies in how urologists address sexual dysfunction risks with patients.

When considering international guidelines, such as those from the European Association of Urology (EAU) and the American Urological Association (AUA), there is a growing emphasis on patient-centered care and shared decision-making in the management of LUTS/BPH. These guidelines generally recommend that clinicians should discuss potential side effects of treatments, including sexual dysfunction, in a clear and understandable manner before initiating therapy [16,17]. Our findings indicate that while many urologists in Saudi Arabia do discuss these risks, the variability in frequency suggests that the consistent application of these principles may need strengthening within this specific context. For instance, the EAU guidelines explicitly state that patients should be informed about the potential impact of 5-ARIs on erectile function and libido and the risk of EjD with alpha-blockers. Our study aligns with the recognition of these specific risks but highlights that the frequency of these discussions is not uniform.

Our findings suggest that while a significant proportion of urologists acknowledge the potential for sexual side effects, there is room for improvement in how these risks are communicated to patients. The variations in discussion frequency across different treatments and the high proportion of “sometimes” or “rarely” discussions indicate that consistent and comprehensive patient counseling is not always achieved. The finding that a considerable number of urologists do not “always” discuss alternative treatments, despite acknowledging the risks of sexual dysfunction, is particularly concerning.

These observations align with findings from Seftel et al. (2007), who reported that physicians, including urologists, tend to underestimate the prevalence of sexual dysfunction in men with LUTS/BPH [18]. In their large-scale epidemiological study, they found that 50% of aging men reported ED or EjD, with LUTS being an independent risk factor. Interestingly, Seftel et al. (2007) also noted differences in perceptions between urologists and primary care physicians (PCPs), with PCPs estimating higher rates of sexual dysfunction due to both LUTS/BPH symptoms and medications compared to urologists [18]. This suggests that the underestimation of sexual dysfunction may be a broader issue across medical specialties, not just within urology [18]. In our study, while we did not compare urologists to PCPs, the variations in discussion frequencies and the proportion of “sometimes” or “rarely” discussions suggest that urologists may also be underestimating the impact of sexual dysfunction on their patients. This underscores the need for increased awareness and improved counseling practices to ensure that patients are fully informed about the potential sexual side effects of BPH treatments.

Additionally, in our study, the trend towards more open discussions among non-Saudi urologists in the univariate analysis, although not statistically significant in the multivariate model, warrants further investigation. This finding may reflect cultural differences in communication styles, varying levels of comfort in discussing sensitive topics, or differences in training and educational background. The association between working in private hospitals and more frequent discussions, while not significant in the multivariate analysis, suggests that factors related to practice setting, such as patient demographics, time constraints, and institutional support, may influence communication practices.

While this study focused on urologists’ perceptions and practices, it is crucial to acknowledge the importance of the patient perspective in the discussion of sexual function. Research indicates that patients prioritize good sexual functioning and expect healthcare providers to initiate conversations about maintaining or improving sexual health [19]. This highlights the need for consistent and comprehensive discussions about sexual functioning with all male patients, as emphasized in our findings, and underscores the potential for misalignment between physician practices and patient expectations. Future research should incorporate patient perspectives through surveys, interviews, or focus groups to provide a more holistic understanding of the communication dynamics and identify specific strategies to facilitate patient-centered communication regarding sexual health in the context of LUTS/BPH management. Such studies could explore factors influencing patients’ comfort in discussing sexual issues, their preferences for how information is delivered, and their perceptions of urologists’ communication skills.

This study has several strengths, including its focus on a culturally sensitive topic and the comprehensive assessment of urologists’ practices across various BPH treatments. The use of a structured questionnaire, distributed via Google Forms, facilitated efficient data collection from a diverse group of urologists. However, the current study has several limitations. The cross-sectional design precludes our ability to infer causality, and the small sample size limits the power to identify significant predictors of frequent and open discussions. The reliance on self-reported data introduces the potential for recall and social desirability biases. Additionally, the generalizability of our findings may be limited due to the over-representation of residents (51.3%) compared to consultants and specialists, who typically lead treatment decisions, and the small number of non-Saudi urologists (*n* = 9), which hinders robust analysis of potential nationality-based differences. The substantial proportion of ‘N/A’ responses for less common procedures like PVP and HoLEP (35–39%) suggests lower familiarity or utilization of these treatments among our respondents, potentially skewing results towards communication practices associated with more frequently performed procedures such as TURP. Furthermore, the small sample size contributes to the wide confidence intervals observed, indicating imprecision in some estimates. While we used a lenient *p* < 0.25 threshold for inclusion in the univariate analysis, which could increase the risk of false positives, we mitigated this by proceeding with multivariate analysis to identify independent associations. Future research with larger, more representative samples, including a greater proportion of consultants and specialists, is warranted to validate our findings and explore these factors in more detail.

## 5. Conclusions

In conclusion, our study highlights the importance of consistent and comprehensive discussions about sexual complications with patients undergoing BPH treatment. The variations observed across different treatment modalities underscore the need for tailored patient counseling based on specific risk profiles. Future research should explore the impact of cultural factors, patient preferences, and educational interventions on the quality of sexual health discussions in BPH management.

The findings of this study have significant implications for clinical practice and policy within Saudi Arabia. At the clinical level, our data suggests a need for more standardized approaches to discussing sexual side effects. Urologists could benefit from specific training and resources to facilitate these sensitive conversations effectively. Implementing standardized protocols or checklists for discussing potential sexual complications before initiating LUTS/BPH treatments could ensure that patients receive comprehensive information, aligning with the principles of informed consent and patient autonomy advocated by international guidelines.

From a policy perspective, several avenues can be explored. Firstly, the development and integration of culturally sensitive guidelines for discussing sexual health in the context of urological treatments within Saudi Arabia are crucial. These guidelines should consider the local cultural nuances and patient preferences to ensure effective communication. Secondly, incorporating communication skills training, specifically focused on discussing sensitive topics like sexual dysfunction, into the curriculum for urology residents and continuing medical education programs for practicing urologists could significantly improve current practices. Thirdly, healthcare institutions in Saudi Arabia could adopt policies that promote a patient-centered approach, ensuring adequate time and resources for comprehensive patient counseling. Furthermore, public awareness campaigns, tailored to the Saudi Arabian context, could help destigmatize discussions about sexual health and empower patients to actively engage with their healthcare providers regarding potential treatment side effects.

The potential impact of these changes is substantial. Improved communication can lead to better patient satisfaction, increased adherence to treatment plans, and enhanced overall quality of life for men undergoing LUTS/BPH management. By aligning clinical practices with international standards and implementing culturally relevant policies, the healthcare system in Saudi Arabia can better address the holistic needs of patients and promote more informed and collaborative healthcare decision-making. Future policies might also consider integrating patient feedback mechanisms to assess and improve the quality of sexual health discussions in clinical practice.

## Figures and Tables

**Table 1:** Demographic and practice characteristics of urologists working in public and private hospitals and clinics across Saudi Arabia.

Professional Status	Frequency	Proportion (%)
Consultant	14	18.4
Fellow	6	7.9
Specialist	17	22.4
Resident	39	51.3
Nationality		
Saudi	67	88.2
Non-Saudi	9	11.8
Region		
Central Region	43	56.6
Eastern Region	11	14.5
North Region	5	6.6
South Region	9	11.8
Western Region	8	10.5
Patients Per Clinic		
<10	13	17.1
15–10	20	26.3
>15	43	56.6
Working Institution		
Public Hospital	36	47.4
Education–Research Hospital	29	38.2
Private Hospital	11	14.4
LUTS/BPH Cases per Month		
0–5	11	14.5
6–15	19	25
16–30	16	21.1
Over 30	30	39.5
Treatment Options for LUTS/BPH		
Alpha-blockers and/or 5-ARIs	76	100
TURP	53	69.7
PVP	16	21
HoLEP	21	27.6
Rezum	35	46

TURP: Transurethral Resection of the Prostate; 5-ARIs: 5-Alpha Reductase Inhibitors; HoLEP: Holmium Laser Enucleation of the Prostate; PVP: Photoselective Vaporization of the Prostate; Rezum: Rezūm Water Vapor Therapy; LUTS: Lower Urinary Tract Symptoms; BPH: Benign Prostatic Hyperplasia.

**Table 2:** Discussion of sexual dysfunction risks by urologists before prescribing treatment.

Discussion of ED with Patients Before Prescribing Treatment						
Treatment	Always n (%)	Often n (%)	Sometimes n (%)	Rarely n (%)	Never n (%)	N/A n (%)
Alpha-Blockers	33 (43.4)	10 (13.2)	10 (13.2)	5 (6.6)	15 (19.7)	3 (3.9)
5-ARIs	32 (42.1)	18 (23.7)	18 (24.0)	1 (1.3)	24 (31.6)	1 (1.3)
TURP	25 (32.9)	19 (25.0)	18 (23.7)	8 (10.5)	3 (3.9)	3 (3.9)
PVP	11 (14.5)	6 (7.9)	12 (15.8)	11 (14.5)	9 (11.8)	27 (35.5)
HoLEP	2 (2.6)	5 (6.6)	11 (14.5)	12 (15.8)	7 (9.2)	29 (38.2)
Rezum	12 (15.8)	9 (11.8)	22 (28.9)	10 (13.2)	7 (9.2)	16 (21.1)
**Discussion of EjD with Patients Before Prescribing Treatment**						
Alpha-Blockers	50 (65.8)	12 (15.8)	11 (14.5)	0 (0)	1 (1.3)	2 (2.6)
5-ARIs	27 (35.5)	13 (17.1)	13 (17.1)	7 (9.2)	13 (17.1)	3 (3.9)
TURP	50 (65.8)	12 (15.8)	10 (13.2)	2 (2.6)	0 (0)	2 (2.6)
PVP	16 (21.1)	6 (7.9)	15 (19.7)	3 (3.9)	7 (9.2)	29 (38.2)
HoLEP	17 (22.4)	5 (6.6)	12 (15.8)	8 (10.5)	5 (6.6)	29 (38.2)
Rezum	18 (23.7)	9 (11.8)	18 (23.7)	7 (9.2)	8 (10.5)	16 (21.1)

TURP: Transurethral Resection of the Prostate; 5-ARIs: 5-Alpha Reductase Inhibitors; HoLEP: Holmium Laser Enucleation of the Prostate; PVP: Photoselective Vaporization of the Prostate; Rezum: Rezūm Water Vapor Therapy.

**Table 3:** Discussion and counseling on alternative treatments based on sexual dysfunction risks.

Discussion of Alternative Treatments Based on Sexual Dysfunction Risks						
Frequency	Treatment Options					
	Alpha-Blockers n (%)	5-ARIs n (%)	TURP n (%)	PVP n (%)	HoLEP n (%)	Rezum n (%)
Always	31 (40.8)	36 (47.4)	36 (47.4)	18 (23.7)	15 (19.7)	22 (28.9)
Often	13 (17.1)	13 (17.1)	12 (15.8)	5 (6.6)	5 (6.6)	7 (9.2)
Sometimes	18 (23.7)	20 (26.3)	19 (25.0)	15 (19.7)	13 (17.1)	18 (23.7)
Rarely	6 (7.9)	5 (6.6)	4 (5.3)	6 (7.9)	7 (9.2)	7 (9.2)
Never	7 (9.2)	0 (0)	2 (2.6)	5 (6.6)	6 (7.9)	5 (6.6)
N/A	1 (1.3)	2 (2.6)	3 (3.9)	27 (35.5)	30 (39.5)	17 (22.4)
**Alternative Treatment Counseling Based on Sexual Dysfunction Risks**						
Always	36 (47.4)	33 (43.4)	41 (53.9)	16 (21.1)	17 (22.4)	27 (35.5)
Often	14 (18.4)	16 (21.1)	14 (18.4)	9 (11.8)	9 (11.8)	8 (10.5)
Sometimes	16 (21.1)	18 (23.7)	16 (21.1)	17 (22.4)	14 (18.4)	21 (27.6)
Rarely	4 (5.3)	4 (5.3)	4 (5.3)	2 (2.6)	4 (5.3)	1 (1.3)
Never	4 (5.3)	3 (3.9)	3 (3.9)	3 (3.9)	6 (7.9)	4 (5.3)
N/A	2 (2.6)	2 (2.6)	2 (2.6)	29 (38.2)	26 (34.2)	15 (19.7)

TURP: Transurethral Resection of the Prostate; 5-ARIs: 5-Alpha Reductase Inhibitors; HoLEP: Holmium Laser Enucleation of the Prostate; PVP: Photoselective Vaporization of the Prostate; Rezum: Rezūm Water Vapor Therapy.

**Table 4:** Discussion of ED and EjD before starting treatment.

Discussion of ED Before Starting Treatment						
Treatment	Always n (%)	Often n (%)	Sometimes n (%)	Rarely n (%)	Never n (%)	N/A n (%)
5-ARIs	39 (51.3)	19 (25.0)	14 (18.4)	2 (2.6)	1 (1.3)	1 (1.3)
Alpha-Blockers and 5-ARIs	38 (50.0)	20 (26.3)	14 (18.4)	2 (2.6)	1 (1.3)	1 (1.3)
TURP	29 (38.2)	14 (18.4)	16 (21.1)	6 (7.9)	7 (9.2)	4 (5.3)
PVP	15 (19.7)	9 (11.8)	12 (15.8)	5 (6.6)	7 (9.2)	28 (36.8)
Rezum	23 (30.3)	8 (10.5)	14 (18.4)	6 (7.9)	11 (14.5)	14 (18.4)
Alpha-Blockers	23 (30.3)	17 (22.4)	9 (11.8)	7 (9.2)	18 (23.7)	2 (2.6)
**Discussion of EjD Before Starting Treatment**						
**Treatment**	**Always n (%)**	**Often n (%)**	**Sometimes n (%)**	**Rarely n (%)**	**Never n (%)**	**N/A n (%)**
HoLEP	17 (22.4)	7 (9.2)	16 (21.1)	6 (7.9)	4 (5.3)	26 (34.2)
PVP	11 (14.5)	6 (7.9)	17 (22.4)	5 (6.6)	7 (9.2)	30 (39.5)
TURP	43 (56.6)	10 (13.2)	16 (21.1)	0 (0)	2 (2.6)	5 (6.6)
Alpha-Blockers and 5-ARIs	36 (47.4)	13 (17.1)	17 (22.4)	4 (5.3)	4 (5.3)	2 (2.6)
5-ARIs	29 (38.2)	14 (18.4)	16 (21.1)	9 (11.8)	5 (6.6)	3 (3.9)
Alpha-Blockers	45 (59.2)	13 (17.1)	11 (14.5)	3 (3.9)	2 (2.6)	2 (2.6)
Rezum	24 (31.6)	11 (14.5)	14 (18.4)	9 (11.8)	6 (7.9)	12 (15.8)

TURP: Transurethral Resection of the Prostate; 5-ARIs: 5-Alpha Reductase Inhibitors; HoLEP: Holmium Laser Enucleation of the Prostate; PVP: Photoselective Vaporization of the Prostate; Rezum: Rezūm Water Vapor Therapy.

**Table 5:** Predictors of frequent and open discussion about sexual dysfunction.

Predictors	Univariate Analysis				Multivariate Analysis			
	OR	LL	UL	*p*-Value	AOR	LL	UL	*p*-Value
Nationality								
Saudi	1			0.06	1.00			0.09
Non-Saudi	4.58	0.88	23.74		7.28	0.69	76.88	
Profession								
Consultant	1			0.54				
Fellow	0.50	0.07	3.67		NA			
Resident	1.17	0.34	3.96					
Specialist	0.55	0.13	2.31					
Region								
Central	1.00				NA			
Eastern	0.72	0.18	2.84	0.52				
North	5.05	0.52	49.03					
South	1.01	0.24	4.29					
Western	2.11	0.45	9.95					
Patients Per Clinic								
<10	1.00			0.28	NA			
15–10	0.41	0.11	1.46					
>15	0.76	0.18	3.17					
Working Institution								
Education–Research	1			0.03	1.00			
Private	3.68	0.39	35.14		3.14	0.20	50.04	0.25
Public Hospital	0.35	0.13	0.94		0.43	0.12	1.61	
LUTS/BPH Cases per Month								
0–5	1			0.22	1.00			0.38
6–15	0.83	0.18	3.88		0.92	0.07	11.63	
16–30	1.43	0.32	6.46		2.79	0.26	29.68	
Over 30	0.42	0.10	1.71		0.72	0.06	8.83	

OR: Odds ratio; AOR: adjusted odds ratio; LL: lower limit; UL: upper limit; NA: not applicable.

## Data Availability

The dataset(s) supporting the conclusions of this article is included within the article.

## Abbreviations

The following abbreviations are used in this manuscript:

ED	Erectile Dysfunction
EjD	Ejaculatory Dysfunction
BPH	Benign Prostatic Hyperplasia
LUTS	Lower Urinary Tract Symptom
OR	Odds Ratio
AOR	Adjusted Odds Ratio
LL	Lower Limit
UL	Upper Limit
95% CIs	95% Confidence Intervals
NA	Not Applicable
TURP	Transurethral Resection of the Prostate
5-ARIs	5-alpha Reductase Inhibitors
HoLEP	Holmium Laser Enucleation of the Prostate
PVP	Photoselective Vaporization of the Prostate
Rezum	Rezūm Water Vapor Therapy
